# TGStools: A Bioinformatics Suit to Facilitate Transcriptome Analysis of Long Reads from Third Generation Sequencing Platform

**DOI:** 10.3390/genes10070519

**Published:** 2019-07-10

**Authors:** Danze Chen, Qianqian Zhao, Leiming Jiang, Shuaiyuan Liao, Zhigang Meng, Jianzhen Xu

**Affiliations:** 1 Computational Systems Biology Lab, Department of Bioinformatics, Shantou University Medical College (SUMC), No. 22, Xinling Road, Shantou 515041, China; 2 Bio-key Health Technologies Co., Ltd., No.9, Huaqiang, Road, Tianhe District, Guangzhou 510630, China; 3 College of Computer Engineering and Applied Mathematics, Changsha University, No.98 Hongshan Road, Kaifu District, Changsha 410005, China

**Keywords:** third generation sequencing, alternative splicing, noncoding RNAs, rare disease, transcriptome analysis

## Abstract

Recent analyses show that transcriptome sequencing can be utilized as a diagnostic tool for rare Mendelian diseases. The third generation sequencing de novo detects long reads of thousands of base pairs, thus greatly expanding the isoform discovery and identification of novel long noncoding RNAs. In this study, we developed TGStools, a bioinformatics suite to facilitate routine tasks such as characterizing full-length transcripts, detecting shifted types of alternative splicing, and long noncoding RNAs (lncRNAs) identification in transcriptome analysis. It also prioritizes the transcripts with a visualization framework that automatically integrates rich annotation with known genomic features. TGStools is a Python package freely available at Github.

## 1. Introduction

Gene-panel and whole-exome sequencing revolutionized mutation detection of the rare Mendelian disease during the past decade. Recently, accumulated analyses demonstrated that transcriptome analysis also significantly improves diagnostic yield in genetically unresolved cases of rare diseases [1,2,3]. Commercially available third generation sequencing (TGS) platforms, such as Pacific Biosciences (PacBio) and Oxford Nanopore Technologies (ONT) developed novel methods to directly capture the long nucleotide sequences from single molecules [4,5]. Compared to canonical second generation sequencing (i.e., RNA-seq), TGS provides a great potential in isoform discovery and characterization of novel long noncoding RNAs. Both are essential aspects of rare disease diagnostics [6,7]. However, the main drawback of TGS is its higher sequencing error rate, which may produce spurious transcripts [8]. Full length transcripts can be identified by comparing them with known genomic annotations, which are associated with actively transcribed regions [9,10]. To the best of our knowledge, currently no bioinformatics tools are built to automatically find nearby genomic features in order to filter transcripts. In this study, we present TGStools, a package that implements multiple tools to facilitate routine transcriptome analysis, such as isoforms comparison, detecting alternative splicing (AS) pattern and lncRNAs identification.

## 2. Materials and Methods

**Figure 1:**
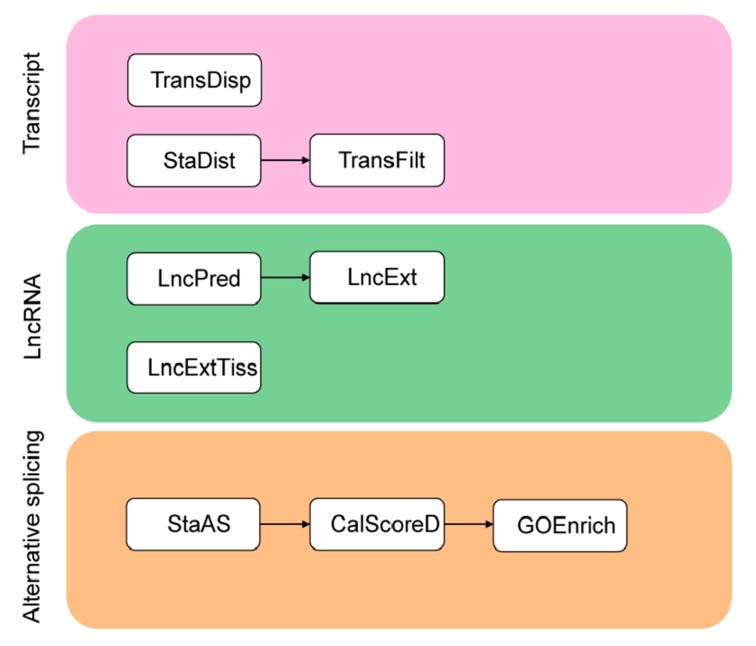
Overview of TGStools. A set of applications to facilitate transcriptome analysis are included in TGStools.

TGStools is a Python package that can be freely obtained from the GitHub project. Test data from both PacBio and ONT platforms, as well as detailed tutorials for each function, is also available online. TGStools includes a set of applications which are classified into three categories (Figure 1). In the ‘Transcripts’ category, the tool ‘TransDisp’ compares the isoforms of the queried gene and displays the sequenced transcripts along with multiple genomic annotations; ‘StaDist’ automatically finds the nearby genomics feature and calculates the distance; ‘TransFilt’ can be used to filter out transcripts according to user-defined distance cutoff. In the ‘LncRNA’ category, the tools ‘LncPred’ and ‘LncExt’ are used to identify non-coding transcripts; ‘LncExtTiss’ extracts tissue-specific lncRNA. Finally, in the ‘Alternative splicing’ category, ‘StaAS’ identifies the alternative events and detects the difference of each alternative splicing event among samples; ‘CalScoreD’ selects the most spliced genes; ‘GOEnrich’ selects top ranked gene ontology terms which are enriched with the most spliced genes. Open access to TGStools at (https://github.com/BioinformaticsSTU/TGStools).

**Figure 2:**
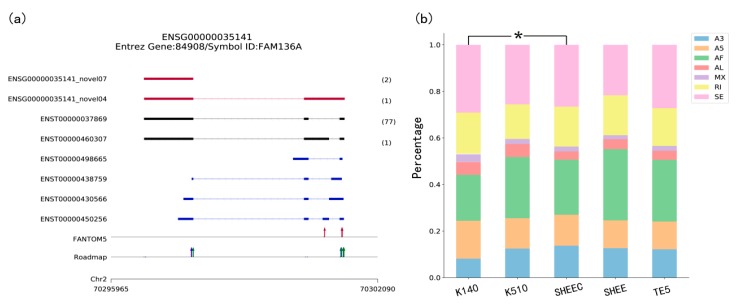
Visualization in TGStools. (**a**) Example of isoforms comparison with known genes and auxiliary annotation. Red track: Novel isoforms from TGS platform; Black track: Known isoforms identified from TGS platform. Blue track: Known transcripts annotation. The numbers of long reads detected are shown in brackets. Red arrow: Known Cap Analysis of Gene Expression (CAGE) promoters identified from FANTOM5 data; in Roadmap track, red, blue and green arrow indicated known H3K4me1, H3K4me3 and H3K27ac marks; (**b**) percentage of splicing events in each sample. The χ^2^ test is used to find the significant difference among samples. Colors indicate different types of AS events. A3: Alternative 3’ splice site; A5: Alternative 5’ splice site; AF: Alternative first; AL: Alternative last exons; MX: Mutually exclusive exon; RI: Retained intron and SE: Skipped exon.

Among the various types of figures TGStools can produce, the transcripts overview plot and the alternative splicing plot are illustrated here (Figure 2). Demonstrations of the other plots can be seen in the Supplementary Material.

## 3. Results

### 3.1. Isoforms Comparison with Known Annotations

The user can import data from the most widely used TGS platforms such as PacBio and ONT after alignment. TGStools includes the latest gene model annotation files from Ensembl (http://grch37.ensembl.org/index.html), the epigenetics marks downloaded from the Roadmap Epigenomics project (http://www.roadmapepigenomics.org/), and the TSS (transcription start site) peaks data generated by the CAGE experiment in the FANTOM5 project (http://fantom.gsc.riken.jp/5/). Since these annotations are typically associated with actively transcribed promoters, the user can identify bona fide full length reads by overlapping transcripts produced from TGS platform with this auxiliary information. TGStools automatically finds the nearby genomic features and produces a summarized report. Given a gene of interest, TGStools also shows the transcript comparison with multiple annotations, from which users can easily identify the spurious transcripts.

The transcript overview plot gives a genome-scale summary along the chromosome location together with known annotation features (Figure 2a). The genomic coordinates of sequenced transcripts are shown in the bottom part of the plot. This is followed by the track which indicates known transcript annotations, whereas known isoforms identified from TGS platform (i.e., Single Molecule Real Time (SMRT) data and ONT data), are shown in black. The numbers of long reads detected are shown in brackets. Comparison of transcription start sites (TSSs) detected in long reads with CAGE promoters and active epigenetic marks are also illustrated at the bottom part of the plot. This figure enables evaluation of whether regulatory elements nearby long transcripts can be detected in other genomic data, in order to eliminate a false discovery. Users can discard some spurious transcripts according to a user-defined cutoff. For example, users can discard the transcript if no genomic features are found upstream or downstream 1 Kbp of its first nucleotide. For an overview of all sequenced transcripts, TGStools also generates distance distributions of TSS in each full length transcript to the closest epigenetic marks and CAGE tags (Supplementary Material, Figures S1 and S2). This plot can be used as an assessment of the overall quality of the sequencing data.

### 3.2. Comparing and Detecting the Shifted Types of Alternative Splicing

Using TGStools, the alternative splicing events can be categorized and illustrated for each sample based on the SUPPA2 algorithm [11]. Users can compare the alternative splicing pattern among different samples with the built-in statistical test. In the alternative splicing plot, different colors indicate the seven AS types. Percentage and event counts of AS types in each sample are illustrated and compared based on the χ^2^ test (Figure 2b and Supplementary Material, Figures S3 and S4). Furthermore, a diversity score is developed to quantitatively measure the isoform usage in each sample (see Supplementary Material and online tutorial). According to user defined cutoffs, the most differentially spliced genes are used to find the significantly enriched functional terms from Gene Ontology. Illustrative plots are also automatically produced for the enriched functional terms (Supplementary Material, Figures S5 and S6).

### 3.3. Finding Tissue Specific Novel Isoforms or lncRNAs

Full length transcripts often encode novel lncRNAs which may be tissue specific. To assist the lncRNA analysis, TGStools can predict the protein coding potential of transcripts using the PLEK and CNCI algorithms, which are commonly used for lncRNA identification [12,13]. Our empirical comparison indicated that the combination of the two software improves the identification of known lncRNAs across the reference catalog (Supplementary Material, Table S1). TGStools generates PLEK and CNCI separate predictions, intersections and union outputs, thus the users can decide on their own. Furthermore, TGStools can compare novel transcripts with the lncRNA reference catalog across human tissues, thus finding tissue-specific novel lncRNAs or isoforms [14]. From the lncRNA Venn plot, users can compare the numbers of identified lncRNAs from different bioinformatics tools (Supplementary Material, Figure S7).

## 4. Discussion

Several large cohort studies revealed that the impacts of splicing pattern, altered expression, as well as non-coding variants contribute to the identification of causal genes, especially for genetically unresolved cases of rare diseases [1,2,3]. We have developed TGStools, which can take input from commonly used long reads platforms, create visualizations to illustrate the full-length transcripts and their expression, and apply functions for analyzing candidate transcripts. TGStools can facilitate researchers in exploring a full-length human transcriptome based on the TGS platform. In the future, we will continuously update TGStools to include user-friendly GUI and more functionalities such as samples classification procedures. Thus, it can also be applied to patient stratification when analyzing clinical datasets [15,16].

## Supplementary Materials

The following are available online at https://www.mdpi.com/2073-4425/10/7/519/s1, Figure S1: Isoforms comparison of queried gene with auxiliary annotation, Figure S2: Distances distribution of TSS in each full-length transcript to the closest epigenetic marks and CAGE tags, Figure S3: Counts of alternative splicing events in each sample, Figure S4: Percentages of alternative splicing events in 5 esophageal squamous cells, Figure S5: Bar plot of Gene Ontology enrichment analysis result, Figure S6: Scatter plot of Gene Ontology enrichment analysis result, Figure S7: Venn plot of lncRNA detected by PLEK and CNCI, Table S1: Comparing the performance of PLEK and CNCI.
