# Supplementary material for: TGStools: A Bioinformatics Suit to Facilitate Transcriptome Analysis of Long Reads from Third Generation Sequencing Platform
Source: Genes (Basel). 2019 Jul 10;10(7):519. doi: 10.3390/genes10070519 (PMC6678717; doi:10.3390/genes10070519)
Supplement: Supplementary file 1 [file genes-10-00519-s001.pdf]

genes

Supplementary Material

1. Introduction

Third generation sequencing can de novo detect long reads of several thousand base pairs, thus provide a global view of the full length transcriptome. It's important to prioritize the results by a visualization framework that automatically integrates rich annotations with known genomic features. *TGStools* is a bioinformatics suit to facilitate routine tasks such as displaying transcripts of gene, characterizing the full-length transcripts and detecting the shifted types of alternative splicing in transcriptome analysis.

2. Tutorial

TGStools is a Python package available at Github (https://github.com/BioinformaticsSTU/TGStools). The installation guide and tutorial can also be found.

3. Application

Previously, we have generated long reads from PacBio SMRT sequencing platform, to investigate the transcriptome-wide heterogeneity and complexity in esophageal squamous cells (Manuscript in revision). Full raw data have been deposited in the Genome Sequence Archive in the BIG Data Center of Beijing Institute of Genomics (BIGD), Chinese Academy of Sciences, under accession numbers (CRA001374).Part of this dataset, together with a published ONT dataset[1],are used for testing the package.

Demonstration 1: Isoforms comparison with known annotation

Users can observe the isoforms comparison with known genes and auxiliary annotation by search of Ensemble Id,Entrez Id and Gene Symbols. As demonstrated blow in **Supplementary Figure S1**, users can compare transcripts obtained from long reads with known transcripts annotation. To evaluate the accuracy of obtained full-length transcripts, users can also compare the transcription start sites (TSSs) detected in long reads dataset with CAGE promoter and active epigenetic marks from Roadmap Epigenomics Project.

TGStools also provide transcriptome-wide assessment of the full length transcripts detected in long reads dataset. **Supplementary Figure S2** indicated the distances distribution of TSS in each full-length transcript to the closest epigenetic marks and CAGE tags, from which users could determine the validity of sequenced full-length transcripts in a sample.

Supplementary Figure S1

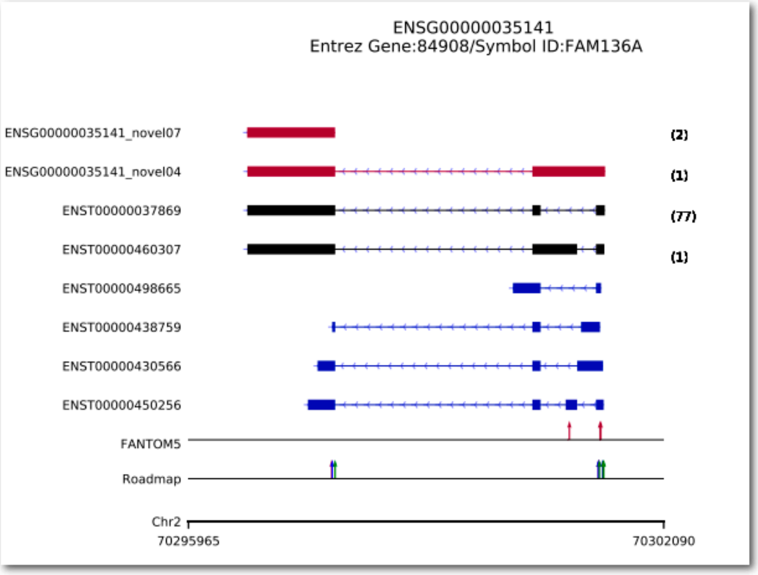

Figure 1. Isoforms comparison of queried gene with auxiliary annotation. This figure shows isoforms of gene ENSG00000035141 and its chromosomal location. Red tracks illustrate novel isoforms from TGS platform (i.e., SMRT data and ONT data); Black tracks for known isoforms identified from TGS platform and blue tracks for known transcripts annotation. The number of long reads detected is shown in brackets. Red arrows indicated known CAGE promoters identified from FANTOM5 data. In Roadmap data, red, blue and green arrows indicate known H3K4me1, H3K4me3 and H3K27ac marks.

Supplementary Figure S2

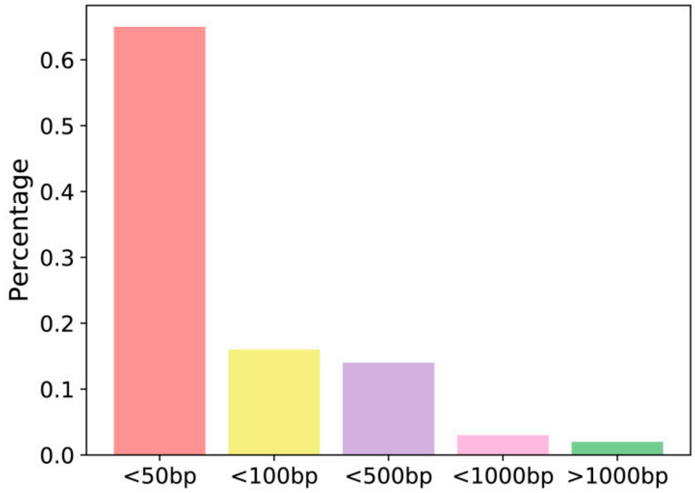

Figure 2. Distances distribution of TSS in each full-length transcript to the closest epigenetic marks and CAGE tags. Pacbio long reads from KSE510 cells are shown. This plot can be used as an assessment of the overall quality of the sequencing data.

Demonstration 2: Alternative splicing analyzing

TGStools analyze alternative events by SUPPA2 algorithm and use Chi-square test to statistically evaluate the proportion of each alternative splicing event in different samples. In addition, *TGStools* produce graphs for both counts and percentage of splicing events in each sample.

Supplementary Figure S3

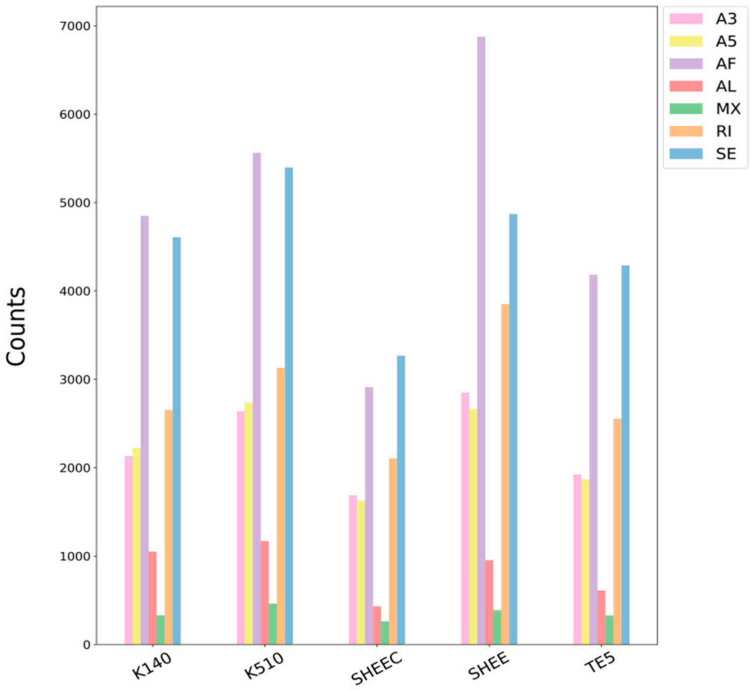

Figure 3. Counts of alternative splicing events in each sample. This figure shows the number of alternative splicing events in each sample. Different types of alternative splicing events are shown with different colors. A3: alternative 3' splice site; A5: alternative 5' splice site; AF: alternative first; AL: alternative last exons; MX: mutually exclusive exon; RI: retained intron and SE: skipped exon.

Supplementary Figure S4

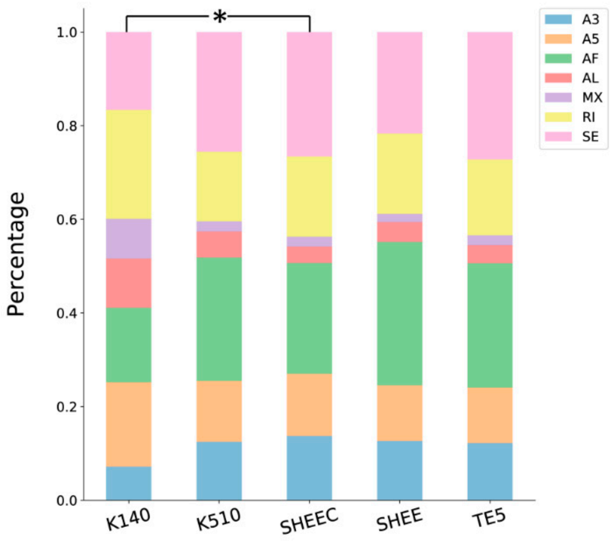

Figure 4. Percentages of alternative splicing events in 5 esophageal squamous cells. This figure shows the percentage of alternative splicing events in each sample. χ2 test is used to find the significant difference among samples. Colors indicate different types of alternative splicing events. A3: alternative 3' splice site; A5: alternative 5' splice site; AF: alternative first; AL: alternative last exons; MX: mutually exclusive exon; RI: retained intron and SE: skipped exon. * $p < 0.05$.

To quantify the differential isoform usage, *TGStools* also employs *D* score to rank the most spliced genes across multiple samples. The score D of each gene is calculated as follows:

$$D_j = \sum_{i=1}^{4} \left(1 - \frac{c_i}{d}\right) \quad \text{whereas} \quad c = a \cap b \,, \quad d = a \cup b \qquad (1)$$

gene ***j*** has isoform set ***a*** , and set ***b*** respectively in cell line X and Y ; ***c*** is the number of isoform intersection for set ***a*** and set ***b***; ***d*** is the number of isoform union for set ***a*** and set ***b***. Thus D sums up scores when comparing the control sample and treated samples. Genes with a higher D value are more diversely spliced. After selecting the top ranked genes, *TGStools* finds the Gene Ontology terms which enriched with the most diversely spliced genes.

Supplementary Figure S5

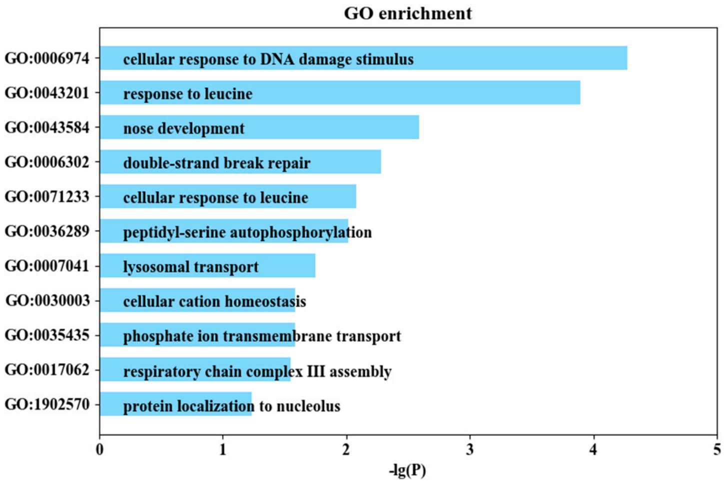

Figure 5. Bar plot of Gene Ontology enrichment analysis result. This figure shows filtered Gene Ontology results sorted by adjusted p-values. Gene Ontology ids and Gene Ontology terms are given on y axis.

Supplementary Figure S6

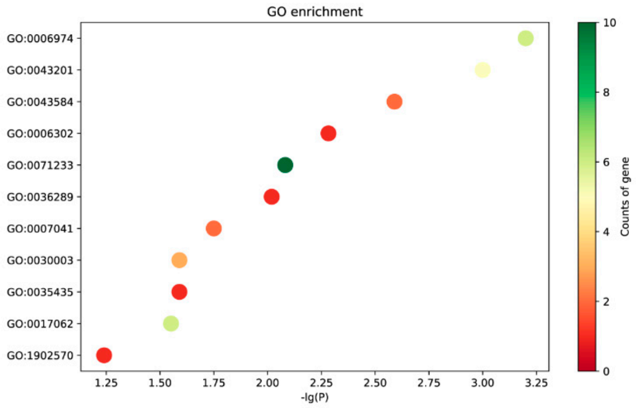

Figure 6. Scatter plot of Gene Ontology enrichment analysis result. This figure shows filtered Gene Ontology results sorted by adjusted p-values. Gene Ontology ids are given on y axis. Significance p value is indicated by x axis whereas the colors of bubbles indicated the number of genes enriched with each GO term.

Demonstration 3: Venn plot of lncRNA detected by prediction tools

Based on two machine learning algorithms, *TGStools* predict lncRNAs from full transcripts. Users can see the individual prediction, intersection and union of the results by Venn plot.

Supplementary Figure S7

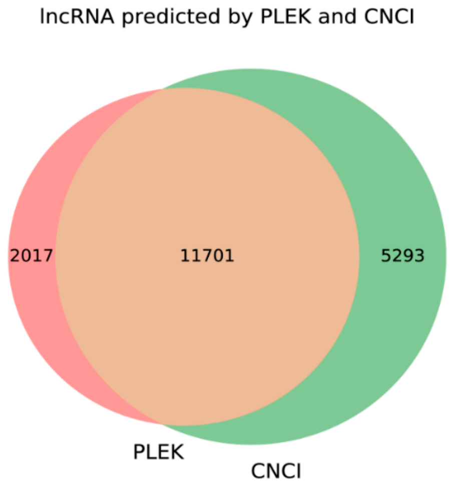

Figure 7. Venn plot of lncRNA detected by PLEK and CNCI. This figure shows the number of lncRNA predicted by PLEK and CNCI.

Supplementary Table S1

Table 1. Comparing the performance of PLEK and CNCI.

Software	PLEK		CNCI		Intersection		Union	
	Coding	LncRNA	Coding	LncRNA	Coding	LncRNA	Coding	LncRNA
GENCODE v29		97.05%		95.41%		91.89%		99.63%
RefSeq release 94	93.99%		84.28%		80.76%		97.50%	

In order to compare the performance of the two software, we ran the test data, in which the RefSeq database (release 94) for human protein-coding transcript and GENCODE v29 for human long non-coding transcripts.

Reference

1. Chuang, T. J.; Chen, Y. J.; Chen, C. Y.; Mai, T. L.; Wang, Y. D.; Yeh, C. S.; Yang, M. Y.; Hsiao, Y. T.; Chang, T. H.; Kuo, T. C.; Cho, H. H.; Shen, C. N.; Kuo, H. C.; Lu, M. Y.; Chen, Y. H.; Hsieh, S. C.; Chiang, T. W., Integrative transcriptome sequencing reveals extensive alternative trans-splicing and cis-backsplicing in human cells. *Nucleic acids research* **2018,** 46, (7), 3671-3691.
